# HTRF: a technology tailored for biomarker determination—novel analytical detection system suitable for detection of specific autoimmune antibodies as biomarkers in nanogram level in different body fluids

**DOI:** 10.1186/s13167-015-0046-y

**Published:** 2015-11-27

**Authors:** Lukas Einhorn, Kurt Krapfenbauer

**Affiliations:** Boehringer-Ingelheim, Dr. Boehringergasse 5-11, A-1120 Vienna, Austria; Institute of Pathophysiology and Allergy Research, Medical University of Vienna, Waehringerguertel 18-20, A-1090 Vienna, Austria; Department of Cranio-Maxillofacial and Oral Surgery, Medical University of Vienna, Waehringerguertel 18-20, A-1090 Vienna, Austria

**Keywords:** Predictive, Preventive, Personalized medicine, HTRF technology, Validation of biomarkers, Immunoassays

## Abstract

**Background:**

Classical methods of gene product analysis such as binding assays (e.g., ELISA, protein chip technology) are generally time-consuming, lab-intensive, less sensitive, and lack high-throughput capacity. In addition, all existing methods used to measure proteins necessitate multiple divisions of the original sample and individual tests carried out for each substance, with an associated cost for each test.

**Method:**

Together with a small biotech company, we developed a new and innovative analytical detection system based on homogenous time-resolved fluorescence (HTRF) technology. Our system facilitates the development of immune assays that measure selective different analytes such as selected biomarkers in a small sample volume at less than 20 min with a much higher sensitivity compared to common binding assay systems such as enzyme-linked immunosorbent assay (ELISA). Recent advances of the application of this novel detection system combine the power of miniaturization, microfluidics, better linear range, and faster quantification.

**Results:**

The power of the HTRF technology offers great promise for point-of-care clinical testing and monitoring of many important analytes such as disease-specific biomarkers in the nanogram level in different human body fluids such as CSF, blood, serum, plasma, and saliva. The linear dynamical range of our HTRF assay was determined between 2.5 and 100 ng/mL. Precision and accuracy calculated for inter- as well as intra-assays was less than ± 10 %. Intra-assay and inter-assay precision for high, medium, and low analyte concentrations show mean CV values less than ± 10 %. Intra- and inter-assay accuracy for all three concentrations show mean recovery values of 80–120 %.

**Conclusion:**

The aim of this work is to describe the development and establishment of this novel HTRF system that allows the very fast detection and quantification of biomarkers in different human body fluids. Furthermore, a specific antibody combination that assures a specific binding of the correct refolded autoimmune IgG is evaluated.

## Overview

The detailed biochemical characterization of possible biomarkers as diagnostic relevant proteins by immunoassay has found utility in diverse applications including biologic biomarker characterization and identification. These applications sometimes require the analysis of correct refolded autoimmune-proteins present in low amounts in biological fluids, or the acquisition of detailed information on the structure of proteins expressed in heterologous systems such blood, serum, plasma, or saliva.

Samples for analysis are routinely generated in aqueous solutions of suspension from different body fluids that may contain a range of non-volatile salts, solvents, albumins, immune globulins, and lipids. These contaminants or matrix proteins can impair the performance of analytical techniques, where they cause a detrimental effect on specificity and sensitivity by interfering with adducts formation, and precipitation or modification. On the other side, immunoassays such as enzyme-linked immunosorbent assay (ELISA) are expensive and time-consuming. ELISA normally requires 4–5 h until the result can be validated. Such ELISA immunoassays for quantification of diagnostic relevant immunoproteins as biomarkers have already been developed and described, but most of them are too unspecific, expensive, and time-consuming. Most of them are using monoclonal capture and/or detection reagents directed against the fragment-constant (Fc) part of the human IgG. However, these assays cannot be used for molecules consisting only of the antigen-binding fragment (Fab) of antibodies or fusion proteins of Fab or a non-antibody protein because these molecules do not contain the epitope on the Fc fragment. Furthermore, the antibodies directed against human Fc exclude the simultaneous use of specific assay reagents which are expressed as Fc fusion proteins to improve stability and expression yields since they cross-react with these reagents. The development of a generic immunoassay for the specific quantification of correct refolded heterodimeric Fab is therefore highly desired. Generally, there exists a great offer of immune assays and it depends on the samples and the required aims which one is the best one. One could be for example ELISA, Western blot, flow cytometry or a protein array, or immunohistochemistry or immunofluorescence assay [1].

### Autoimmune antibodies as selective biomarkers

Antibodies (IgG, IgM, IgA, IgE) are very specific reagents and are the specific cellular part of the immune system. Antibodies are multifunctional molecules that mediate the specific binding to the antigen and cause effector mechanisms like activation of the complement system or signal transduction, so they are an important part of the immune system [2, 3] and can be used as an important tool to determine predictive biomarkers for autoimmune diseases in human body fluids. Every antibody has four chains, two identical light chains and two identical heavy chains that are both connected and stabilized through non-covalent connections and covalent connections like disulfide bridges. The N-terminal end is the variable region and is responsible for the specific antigen binding to the antibody. At the end of the variable region is the hyper variable region or the so called complementary determining region (CDR) which is responsible for the non-covalent binding of antigens. The constant region is responsible for the effector mechanism. So, antibodies are often figured as “Y” because they have a long and a short arm. The junction between them functions as a binding region and is named the Hinge region [2, 4].

Today, most of the monoclonal antibodies are manufactured by fusion of a B cell and a myeloma cell of a mouse. The aim is to combine the attributes of both, the ability to produce specific immunoglobulins and the ability to grow endlessly which make myeloma cells immortal. For the fusion, polyethylene glycol (PEG) is used. Monoclonal antibodies become more and more important as therapeutic agents so that until 2009, already 22 monoclonal antibodies have been approved for use. Also, the fragments of the monoclonal antibodies, especially the Fab, are preferred in therapeutic usage. A polyclonal antibody is not as specific as a monoclonal one, but it is much more sensitive [3–6]. For the preparation of an antibody for a special immune assay, a conjugation of the antibody for example with a special enzyme can be done to get a signal. Favored systems are *biotin* or enzymes like *alkaline phosphate* and *horseradish peroxidase*. Every manipulation can influence the antibody and therefore may change the conformation or the binding of the antigen [3, 7–9].

For a long time, antibody drug designers hypothesized that antibodies could be used to engineer customized and personalized therapeutics with pharmacologic properties. Designed antibodies could confer advantages like better purity and improved quality and quantity of goods. Physiochemical properties of therapeutic molecules can be altered by fragmentation of antibodies. For example, fragments with smaller size are able to penetrate into inaccessible tissues [10].

**Fig. 1:**
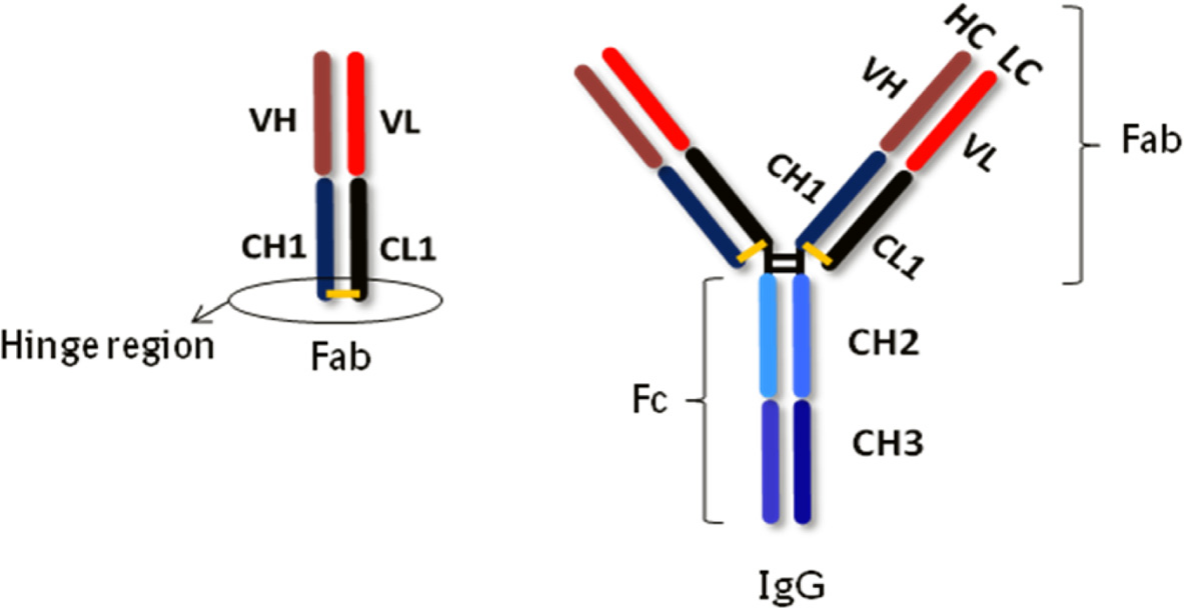
Structure of an antibody and the Fab fragment after digestion with pepsin/papain (taken from [10] and adapted)

**Fig. 2:**
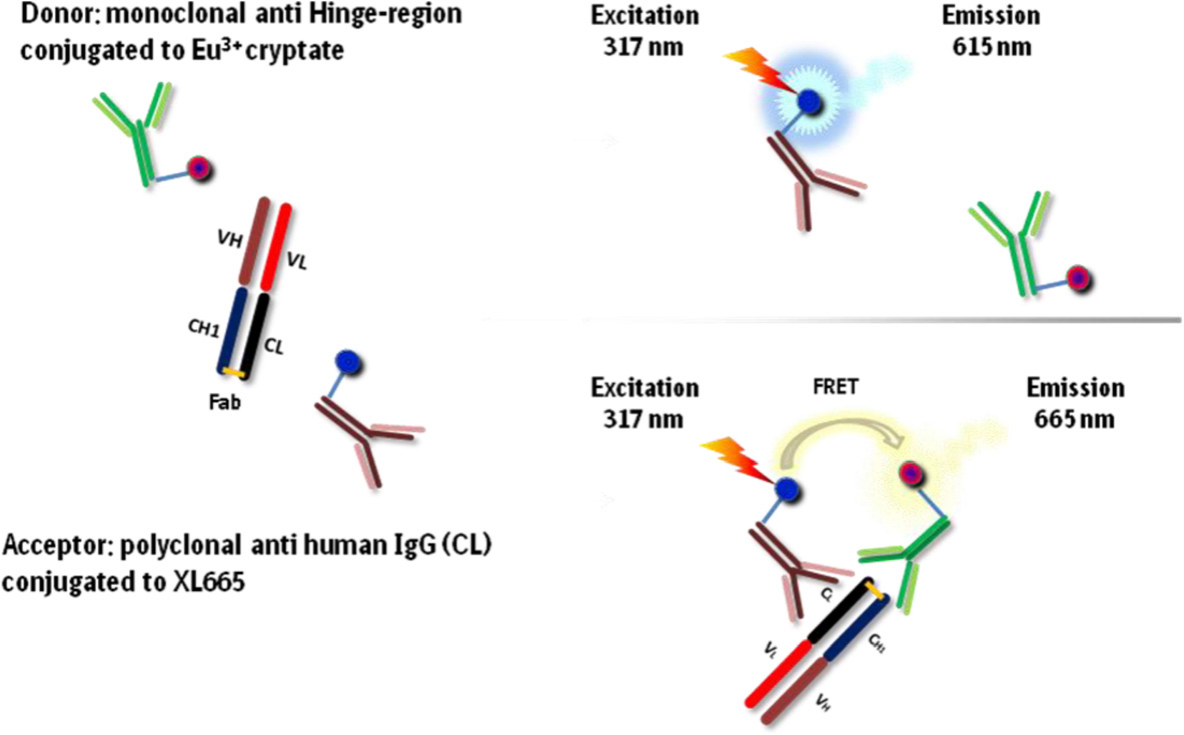
Overview of FRET technology: without and with close proximity between donor and acceptor (taken from [10] and adapted)

If immunoglobulins are digested with papain or pepsin, their amino acid binding can be divorced and three fragments obtained (Figs. 1 and 2). Two of them are equivalent and include the complete light chains that are already associated with disulfide bridges to the CH1 domain of the heavy chains. This is also the reason why these fragments still have the antigen-binding site and so they are called *fragment antigen-binding* (Fab). Fab fragments are the oldest class of monoclonal antibody fragment therapeutics and the most successful one (“accounting 49 % of fragments” that entered clinical development are Fabs). The third fragment consists of the remaining heavy chains that are connected with disulfide bridges. This part is specific for each single isotope, constant, and also independent from the antibody specificity. It is named *fragment-constant* region (Fc region). The antibody fragments have the advantage of a similar binding specificity like the full size immunoglobulin [2, 4, 10].

Fab fragments have only a part of the constant heavy chain and that is why protein A and G are not able to bind, which make Fab proteins a possible tool for ELISA assays. Sometimes, negative side effects can occur because of effective ranges of the Fc fragment which are eliminated by using only the Fab one. Furthermore, the smaller size of antibody fragments allows a faster and deeper penetration of tissues and tumors. The half-life of the fragments can be increased as the half-life of the whole antibody by covalent modifications with polyethylene glycol or other large structures [11–14].

For example, the following fragments are used in therapy: abciximab (ReoPro, Centocor/Johnson & Johnson) is a Fab fragment of a chimeric antibody against platelet glycoprotein IIb/IIIa, approved in 1994 as an adjunct to prevent thrombosis during coronary artery catheterization for ST-elevation myocardial infarction. Ranibizumab (Lucentis, Genentech) is a humanized Fab directed against vascular endothelial growth factor A, approved in 2006 as a treatment for neovascular (wet) age-related macular degeneration. Certolizumab pegol (UCB) is a pegylated anti-TNFα Fab approved in 2008 for treatment of Crohn disease [10].

### Homogeneous time-resolved fluorescence technology

In the 1990s, the company Cisbio Bioassays, specialized on immunoassays, invented homogenous time-resolved fluorescence (HTRF) assay, a specialized immunoassay. HTRF assay combines fluorescence resonance energy transfer (FRET) technology with time-resolved (TR) measurement to be an ideal platform for drug target studies used in high-throughput methods [11, 12].

FRET (Fig. 2), also named Förster resonance energy transfer, use the effect of energy transfer between two fluorophores, a donor and an acceptor, when they are in close proximity as for example when both are binding to a different part of an analyte. Therefore, each partner has to be coupled with a fluorescent label. The energy transfer from the donor to the acceptor takes place by an excitation of the donor by an energy source (e.g., flash lamp) that in close proximity triggers an energy transfer towards the acceptor that emits specific fluorescence at a given wavelength [11–13]. FRET systems are characterized by Förster’s radius distance with a FRET efficiency of 50 %, and for HTRF, the radius is dependent on the used acceptors between 50 and 90 Ǻ [12].

**Fig. 3:**
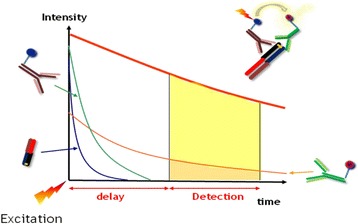
Time-resolved measurement of emission caused by energy transfer from donor to acceptor (taken from [10] and adapted)

The donor-acceptor complex can be detected without the need for physical separation from the unbound components. That is why it is a homogeneous assay without the need for further separation or washing steps. Another advantage of the HTRF assay is the time-resolved measurement (Fig. 3) because some sample components like buffers, proteins, and cell lysates influence the results with a negative background that is transient (lifetime in the nanosecond range) and that can be eliminated with time-resolved measurement. The introduced time delay of approximately 50 to 150 μs between excitation and measurement allows a clearance of all unspecific short-lived emissions. Contrary, acceptors emit long-lived fluorescence and therefore long-lived emissions [11–13] (Fig. 3).

**Fig. 4:**
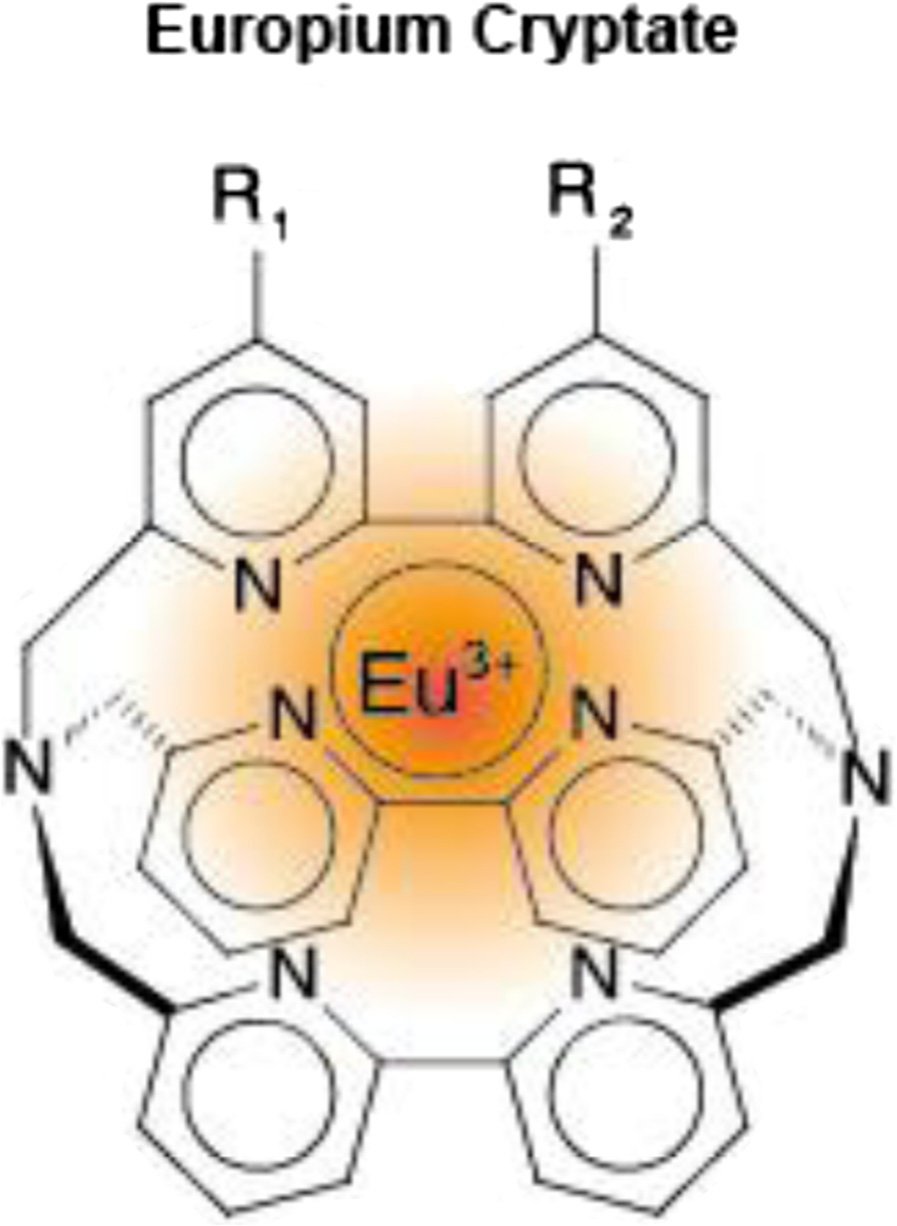
Europium cryptate structure typically consists of a tris bipyridine macrocycle in which the lanthanide ion is tightly embedded (taken from [10] and adapted)

Fluorescent partners have to fulfill multiple criteria. The emission spectra must show non-overlapping regions to measure each partner individually. A high quantum yield and a fluorescence emission within a region of the spectrum remote from that naturally produced by proteins are preferred. So, a combination of different fluorophors is used. The donor consists of a rare earth complex in which the lanthanide ion (europium or terbium) is attached in a macrocycle that distributes robustness and long-lived fluorescence properties. Europium cryptates (Eu^3+^ cryptate; Fig. 4) developed by Prof. J.M. Lehn is a series of rare earth complexes whose macrocycle is based on an embedded trisbipyridine motif. This structure allows a transfer and collection of energy to Eu^3+^ which releases this energy in a specific fluorescent pattern that can be measured at 665 nm. The first acceptor was XL665, a phycobiliprotein pigment purified from red algae that is a heterohexameric form of 105 kDa cross-linked for better stability and preservation of its photophysical properties. The now used acceptors (d2) are similar to XL665, but 100 times smaller, displaying a series of photophysical properties close to XL665. The excitation spectra overlap those of EU^3+^ cryptate emissions, and so, it is allowed to excite from donor to acceptor whose maximum emission of 665 nm spans a region where HTRF cryptates do not emit [11, 12].

Long signal stability is another benefit that includes read time flexibility, the ability to perform kinetic studies and increased sample compatibility. The robustness can be explained by the photophysical and chemical stability of the involved fluorophores. The cryptate structures resist harsh conditions or additives, and the europium cryptate resistance can be enhanced by adding fluoride ions. Another positive property of cryptates is that they do not photo bleach (the disappearance of fluorescence emission after repeated excitation) and that the acceptors are compatible with a wide range of conditions [11].

For measuring two emissions, wavelengths are used, 620 and 665 nm, which allow the ratiometric reduction of data that can reduce well-to-well variations. It allows the measurement of the donor and the acceptor wavelength whereby donor emission at 620 nm is taken for internal reference, while acceptor emission at 665 nm is used as indicator of the biological reaction being assessed [12].

Summarized, HTRF assay is an immune assay that combines homogeneity, time-resolved measurement without interference of other components, the possibility of automatization because there is no need for washing or separation steps and the FRET technology. Overall, HTRF is a highly specific, robust technology for the detection of molecular interactions and is widely used for drug development; with a standard curve, quantification is also possible [12].

### Sources of error

All existing immune assays share the sensitivity to interferences. That can be cross reactions, unspecific bindings, matrix effects, and interactions between the analyte and antibodies with disrupting substances. Fluorescence-labeled antibodies are further influenced by interactions between proteins of the sample and the fluorescence dye. Labeling with enzymes can lead to unspecific bindings, and the immobilization may lead to structural changes [14].

*Interference because of unspecific labeling* can be induced by unspecific binding of the labeled antibody to the surface. This problem could be avoided by blocking the surface before detection [14]. Furthermore, the use of the wrong blocking buffer or the inadequate blocking process leads to unspecific bindings. Fluorescent dyes can change the binding properties, that leads to an increased surface, catching of an antibody or foreign protein, and consequently to a wrong positive measurement signal or high background [15].

Another source of interference could be the *choice of antibodies*. Generally, the quality of highly purified antibodies should be guaranteed by the manufacturer. Especially for sandwich ELISA, it should be ensured that the secondary antibody (for example *goat anti-rabbit-peroxidase-labeled*) does not bind to the analyte [14, 15].

*Matrix effects* are defined as the sum of the whole number of effects of all sample components that influence the measurement of the target analyte. Causes of risk are sample proteins that are candidates for unwanted interactions with the analyte or the antibodies [14].

Interferences because of *chemicals and buffers* are an important, but often ignored, point. Viscosity, salt concentration, and pH directly influence binding between analyte and antibody. Antibody bindings normally prefer physiological conditions at about pH 6–8 and a NaCl concentration of about 150 mM. Furthermore, it is beneficial if the matrix of standard and sample is the same [14].

Further problems can occur with *cross reactions and unspecific bindings*. Cross reactions are defined as the ability of an antibody to bind more than one target structure. The cross-reacting agent is known. Unspecific binding describes the binding of a substance that is available with a higher concentration compared with the target analyte [14].

*Proteins and endogenous parts of proteins* may also influence the measurement. Natural proteins like *albumin* or *lysozyme* have the ability to bind other proteins so no further connection with antibodies is possible. Unspecific bindings and cross-contaminations are the consequences. Endogenous proteins can mask the analyte or interfere the binding with an antibody. To determine the stability of the sample, many conditions have to be considered like the sample taking or the age of the sample. The storage conditions, temperature sensitivity, and the repeated freezing and thawing of the sample are further important points for interferences and should be investigated [14, 15].

Finally, interferences because of *human antibodies against immunoglobulins of animal origin* and *heterophile antibodies* which have false positive signals as consequence have to be mentioned [15].

So, there exist many obstacles which can influence an immune assay. To reduce interferences, they have to be identified and the reasons have to be detected. Finally, an appropriate strategy has to be established to optimize the assay as good as possible [14].

### Hook effect

**Fig. 5:**
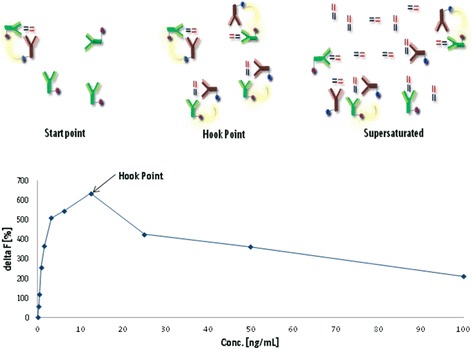
*Hook* effect and consequences (taken from [10] and adapted)

The *Hook* effect (Fig. 5) is a false negative result. The effect appears in highly concentrated samples which are mixed directly with the assay antibodies (for example used by the HTRF assay). In that case, the high concentrations of the analyte, which exceed the concentration of the used assay antibodies, saturate both antibodies (started at the so named *Hook* point). The high concentrations simulate a lower concentration that leads to an underestimation of the true value. To avoid the influence of *Hook* effect and its impact on the results, higher concentrations of the assay antibodies or a higher dilution of the used samples can be helpful [15].

### Steps for evaluation of the HTRF immune assay

From the first development of an assay to the continuous use, the degree of stringency required in data analysis was underestimated, and consequently, many publications with questionable results and quality have been issued. With the ICH guidelines (ICH-Q2A and ICH-Q2B), a standardized and exact characterization and validation guideline was introduced [16].

In the course of characterization and validation studies, a number of key issues in research and development must be implemented. “*The complexity of an analytical validation should reflect the aim of the analysis and thus has to be in accordance with the intended use*.” So, validation is comparable with a demonstration that a method can fulfill its designated duty [16].

Specificity, selectivity, accuracy, precision, recovery, calibration curve, and stability of analyte spiked in samples are some examples that are performed during this work.

Specificity “*is the ability to assess unequivocally the analyte in the presence of components which may be expected to be present*”. With the specificity, it is possible to assure the correct detected analyte and the difference between the test method value and the theoretical value can be described. It is a combination of identification tests, assay, and impurity tests [16, 17].

Selectivity “*is the ability of an analytical method to differentiate and quantify the analyte in the presence of other components in the sample* [18].” The selectivity is determined with Western blot and a specific antibody.

Accuracy “*describes the closeness of mean test results obtained by the method to the true value (concentration) of the analyte. Accuracy is determined by replicate analysis of samples containing known amounts of the analyte* [18].” The accuracy is comparable with the calculated recovery results of standard curves done by ELISA and HTRF experiments. “*The mean value should be within 15% of the actual value except at LLOQ, where it should not deviate by more than 20 %* [18].”

Precision “*describes the closeness of individual measures of an analyte when the procedure is applied repeatedly to multiple aliquots of a single homogeneous volume of biological matrix* [18].” Here, the precision coefficient of variation [%] is measured with different dilutions in triplicate and is done with different concentrations with ELISA and HTRF assay. “*The precision determined at each concentration level should not exceed 15 % of the coefficient of variation (CV) except for the LLOQ, where it should not exceed 20 % of the CV* [14].” So, the precision describes the assay variability and expresses the closeness of agreement between many measurements from multiple sampling of the same sample [16].

Recovery “*is the detector response obtained from an amount of the analyte added to and extracted from the biological matrix, compared to the detector response obtained for the true concentration of the pure authentic standard*.” “*Recovery of the analyte need not be 100%, but the extent of recovery of an analyte and of the internal standard should be consistent, precise, and reproducible*.” The acceptable range of recovery is limited to ±20 % of 100 % [18].

Calibration/standard curve: “*A calibration (standard) curve is the relationship between instrument response and known concentrations of the analyte* [18].” For each blot or plate, a separate standard curve is produced in order to compare the results of the analyte with the standard using the same conditions (see 4.2 and 4.3). “*The lowest standard on the calibration curve should be accepted as the limit of Quantification*” (LLOQ) [16, 18]. So a range for linearity, evaluated by visual inspection of a plot of signals as a function of analyte concentration, with a *R*^2^ > 0.98, is established. The acceptable range between an upper and lower quantitation limit (QL), that is the highest and lowest possible measurements with an acceptable precision and accuracy, depends on the intended application of the procedure [16, 17].

Stability: “*Drug stability in a biological fluid is a function of the storage conditions, the chemical properties of the drug, the matrix, and the container system* [18]”. The standard with known concentration is spiked in samples to test the influence of matrix effects and the consequential stability. Therefore, the recovery is calculated and compared with the known standard concentrations.

## Methods

Homogenous time-resolve fluorescence (HTRF) assay combines fluorescence resonance energy transfer (FRET) technology with time-resolved (TR) measurement. Differently to an ELISA assay, no immobilization, washing, or separation is necessary. So, for a HTRF, only three compartments are necessary, the analyte, the donor antibody with Eu^3+^ cryptated, and the acceptor with d2, an enhancement of XL665 cryptate. They can be mixed in the well of the microtiter plate, and after a short incubation time of a few hours, the detectable emission caused by an energy transfer from donor to acceptor is detectable and stable over a long period of time [12] (Table 1).

**Table 1:** Antibodies used for HTRF

Manufacturer/name	Specific for	Format	Labeled as	Catalog number
Abcam	Correct refolded Hinge region	Mouse monoclonal [2A11] to human IgG	Donor: conjugated to Eu^3+^ cryptate	Ab7497
Novus	Light chain	Goat anti-human IgG antibody; polyclonal	Acceptor: conjugated to d2	AP003CUS01
Novus	Light chain	Goat anti-human IgG antibody; polyclonal	Donor: conjugated to Eu^3+^ cryptate	AP003CUS01

The monoclonal *Abcam* antibody and the polyclonal antibody *Novus* are used as donors labeled with europium cryptate (Eu^3+^) that allows a collection and transfer of energy to Eu^3+^ which releases this energy in a specific fluorescent pattern that can be measured at 620 nm. As acceptor, polyclonal *Novus* antibody is used, marked with d2 that possesses a series of photophysical properties similar to XL665 with 100 times smaller organic structure and is measured at 665 nm.

The dilution buffer is used to dilute the samples and prepare the standard curve. The conjugate buffer is used to prepare the HTRF conjugates (Tables 2, 3, 4, and 5).

**Table 2:** Ordernumber from CisBio of the antibodies and buffers used for the experiments

	mAb2- Eu3+ cryptate	pAb1-d2	Dilution buffer	Conjugate buffer
Stock solution	211μg/mL	497μg/mL	–	–
Ref. # (Cisbio)	64CUSKAYE	64CUSDAYE	62DL3DDD	62RB3RDD

**Table 3:** Further reagents used for HTRF

Reagents	Manufacturer
HQ water	In-house
1 M Sodiumchlorid (NaCl)	Merck
Drug substance proteinX [1 mg/mL]	BIRCV/BPA PSDP
Drug substance protein [4.3 mg/mL]	BIRCV/BPA PSDP
1x TBS-casein block/diluent	BioFX Laboratories
Tween 20	Sigma Aldrich

**Table 4:** Dilution factor to prepare the working solution from stock solution and concentration of the working solution

	Dilution factor to prepare the working solution from stock solution	Concentration of the working solution	Conjugate final concentration in the well
pAB1-Eu^3+^	576	0.34 ng/μL	0.085 ng/μL
pAb1-d2	124	4 ng/μL	1 ng/μL
mAb2- Eu^3+^	517	0.408 ng/μL	0.102 ng/μL

**Table 5:** Reagent preparation, as prepared in the best pair work of CisBio [10]

	Dilution factor to prepare the working solution from stock solution	Concentration of the working solution	Conjugate final concentration in the well
pAB1-Eu^3+^	576	0.34 ng/μL	0.085 ng/μL
pAb1-d2	124	4 ng/μL	1 ng/μL
mAb2- Eu^3+^	517	0.408 ng/μL	0.102 ng/μL

### Working procedure

The HTRF analysis involves four steps:

1. Dilution of the samples with dilution buffer (it is also possible to use 1xTBST buffer for dilution to compare with ELISA assay)
2. Dilution of the antibodies with reconstitution buffer
3. Mixing of the antibody and the sample in the wells and incubation for ~20 min
4. Measuring the plate

For 384 plates:

- Use 384 low volume white plate
- Add 10 μL sample or diluent buffer for negative control
- Add 5 μL pAb1-d2 conjugate
- Add 5 μL mAb2-Eu^3+^ cryptate conjugate/pAb1-Eu^3+^ cryptate conjugate
- Incubate at room temperature
- Measuring: in the period of time at 317 nm (reference 665 nm/620 nm) with Tecan Safire 2 plate reader

It is possible to shorten the working process by mixing the two antibodies before adding them to the well (Tables 6 and 7). For measuring, Safire 2 from Tecan is used with the Magellan Software (Tecan version 7.1) for evaluation. Two sequential measurements should be carried out: at 620 nm for the cryptate emission and at 665 nm for the specific signal emitted by the acceptor (XL665 or d2). The ratio of the two fluorescence intensities 665/620 (acceptor/donor) enables the calculation of Delta F [%] which represents the relative energy transfer rate for each sample. Safire 2™ readers must be appropriately be configured for HTRF readout by setting up the measurement conditions in the “multilabeling” function of Magellan software. In particular, these parameters should be entered as below. The reader only allows high performance for the used HTRF measurement when the HTRF is run with white plates. No special upgrade is required for HTRF readout, as it is a monochromator-based instrument.

**Table 6:** Volume of antibodies and samples for each plate

	Volume per plate	pAb1-Eu^3+^ conjugate per well [ng]	pAb1-d2 conjugate per well [ng]	mAb2-Eu^3+^ conjugate per well [ng]
96 regular plates	200 μL	17	200	20.4
384 small volume plates	20 μL	1.7	20	2.04

**Table 7:** Volume of antibodies and samples for each well

	Final volume per well	Volume of sample per well	Volume of d2 conjugate per well	Volume of Eu3+ cryptate conjugate per well
96 regular plates	200 μL	100 μL	50 μL	50 μL
384 small volume plates	20 μL	10 μL	5 μL	5 μL

**Table 8:** Comparison of the final results of ELISA and HTRF assay

	ELISA		HTRF	
	proteinX	proteinY	proteinX	proteinY
Linear range	36–1 ng/mL	10–1 ng/mL	100–2.5 ng/mL	50–3.6 ng/mL
Lower limit of detection (LLOD)	2.5–1 ng/mL	1 ng/mL	3.6–2.5 ng/mL	3.6 ng/mL
Upper limit of detection (ULOD)	36 ng/mL	10 ng/mL	>100 ng/mL	50 ng/mL
Inter-assay precision	5.71–9.9 % (*n* = 6)	5.9–11.4 % (*n* = 6)	5.7–16 % (*n* = 6)	4.3–13.4 % (*n* = 6)
Inter-assay accuracy	87.9–99.4 % (*n* = 6)	80.9–95.9 % (*n* = 6)	90–93 % (*n* = 6)	87.5–98.1 % (*n* = 6)
Intra-assay precision	2.1–3.7 % (*n* = 3)	8.1–10.3 % (*n* = 3)	3.3–9.5 % (*n* = 3)	2.9–10.7 % (*n* = 3)
Intra-assay accuracy	92.9–97.9 % (*n* = 3)	88–95.9 % (*n* = 3)	101–105 % (*n* = 3)	96.6–99.2 % (*n* = 3)

Measurement 1

**Table:** 

Excitation wavelength	317 nm
Excitation bandwidth	20 nm
Emission wavelength	620 nm
Emission bandwidth	10 nm
Number of reads	100
Lag time	60 μs
Integration time	500 μs

Measurement 2

**Table:** 

Excitation wavelength	317 nm
Excitation bandwidth	20 nm
Emission wavelength	665 nm
Emission bandwidth	10 nm
Number of reads	100
Lag time	60 μs
Integration time	500 μs

### Results of homogenous time-resolved fluorescence assay

Actually, the development of the HTRF assay is similar to the ELISA development.

For development, the following experiments have been performed:

- Restriction of the detection range for different standards and definition of the linear range and the preferred incubation time
- Accuracy and precision of the linear range
- Comparison of the possible antibody pairs for the specific determination of the biomarker as target protein
- Measuring of different biomarker proteins in different concentrations and comparison with ELISA results
- HTRF spike-in experiments to determine possible matrix effects
- Some experiments to improve the HTRF assay and to minimize *Hook* effect

With the results of the measured wavelengths, calculations can be done (S = standard variance; M = mean of delta OD; k = gradient; d = *y*-axis intercept). First, the ratio of the two raw data signals (rd) is calculated:

$$ \begin{array}{c}\hfill \mathrm{ratio}=\frac{{\mathrm{rd}}_{650 nm}}{{\mathrm{rd}}_{620 nm}}\ast 10000\hfill \\ {}\hfill \varDelta F=\frac{\mathrm{mean}\kern0.5em {\mathrm{ratio}}_{\mathrm{sample}}-\mathrm{mean}\kern0.5em {\mathrm{ratio}}_{\mathrm{negative}}}{\mathrm{mean}\kern0.5em {\mathrm{ratio}}_{\mathrm{negative}}}*100\hfill \end{array} $$

So, the calculated fluorescence ratio Δ*F* [%] represents the time-resolved fluorescence of the aim energy transfer from donor to acceptor antibody.

$$ \begin{array}{c}\hfill \mathrm{Concentration}\kern0.5em \mathrm{per}\kern0.5em \mathrm{well}\left[\frac{\mathrm{ng}}{\mathrm{Well}}\right](c)=\frac{M-d}{k}\hfill \\ {}\hfill \mathrm{Sample}\kern0.5em \mathrm{concentration}\left[\frac{\upmu \mathrm{g}}{\mathrm{mL}}\right]=\frac{c*\mathrm{dilution}\kern0.5em \mathrm{factor}}{1000}\hfill \\ {}\hfill \mathrm{Recovery}\left[\%\right]=\frac{\mathrm{sample}\kern0.5em {\mathrm{concentration}}_{\mathrm{calculated}}}{\mathrm{sample}\kern0.5em {\mathrm{concentration}}_{\mathrm{theoretical}}}*100\hfill \end{array} $$

Restriction of the detection range for different standards and definition of the linear range and the preferred incubation time.

For the determination of a steady standard curve of standard proteinX and standard protein, an antibody combination of the monoclonal antibody *Abcam* labeled with europium (Eu^3+^) cryptate and specific to the Hinge region as donor and as second antibody the polyclonal *Novus* labeled with d2 and binding the light chain as acceptor is used. The sample and antibody mix volume (=5 μL donor and 5 μL acceptor antibody) is 10 μL.

**Fig. 6:**
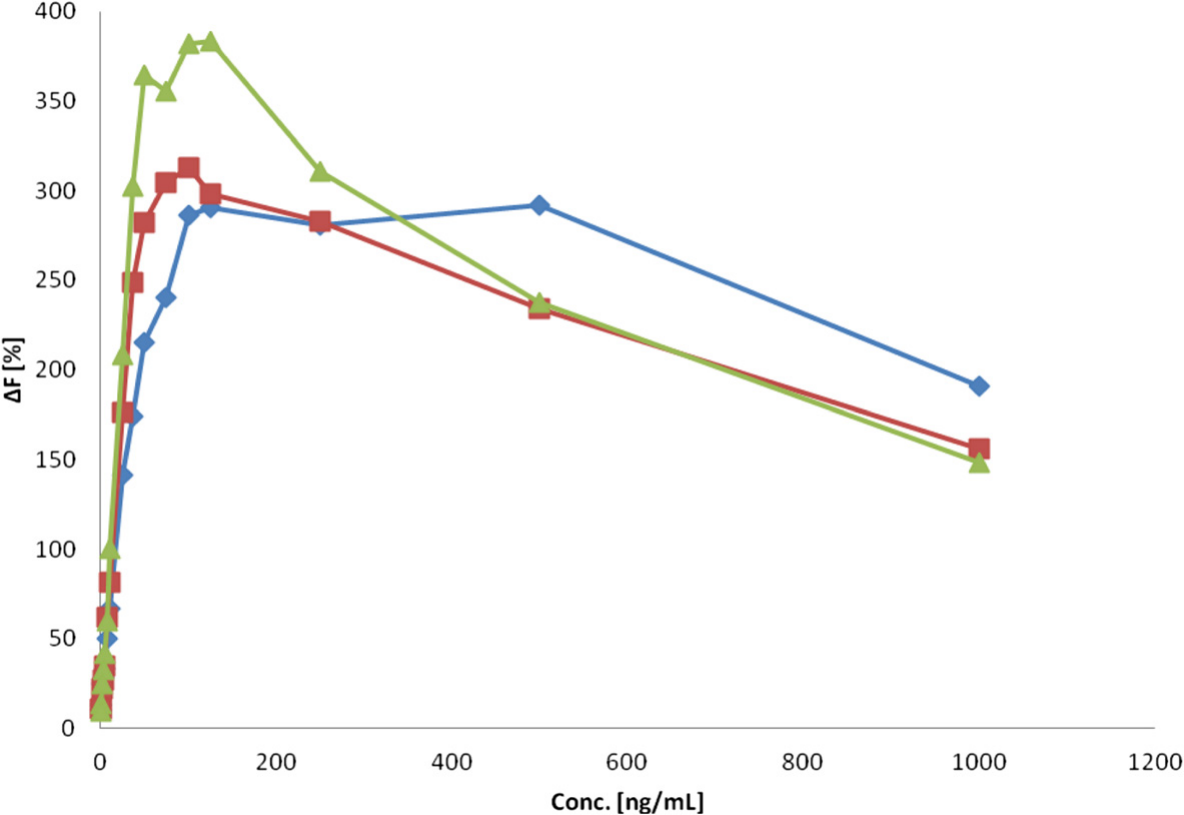
Standard proteinY with different concentrations and different incubation times; standard curve after 1 h (*diamond*), standard curve after 2 h (*square*), standard curve after 3 h (*triangle*)

**Fig. 7:**
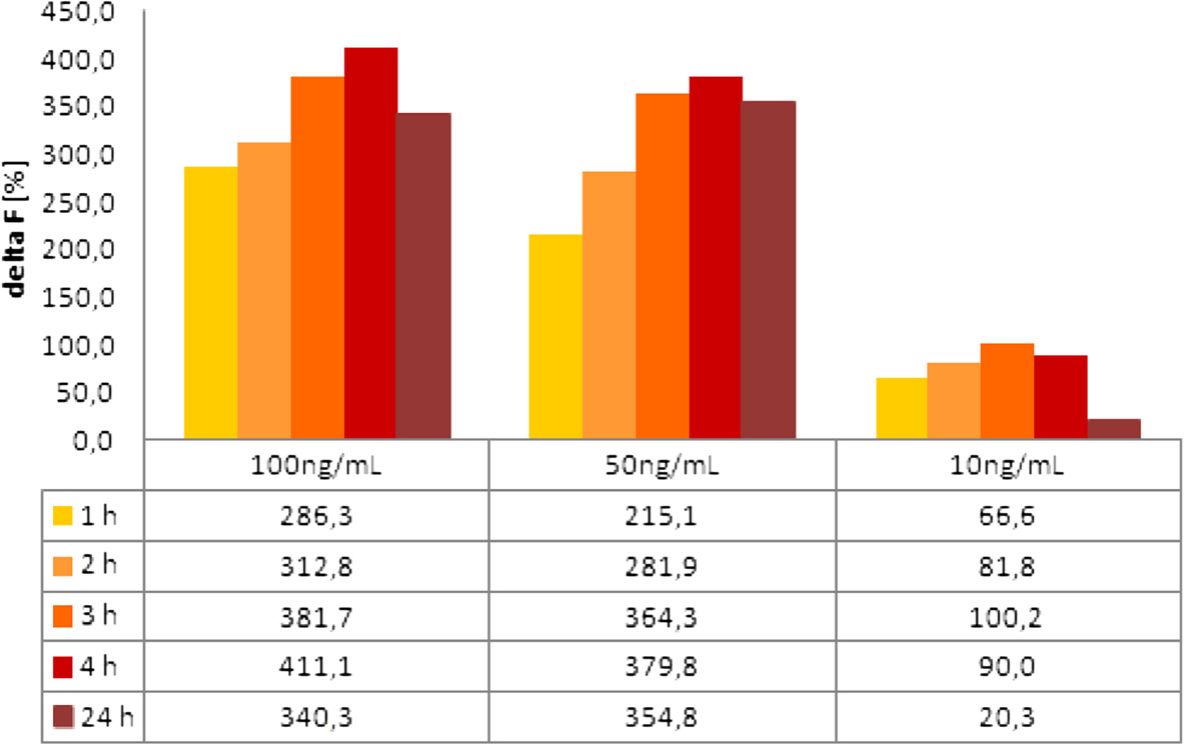
Different incubation times for proteinY using three different concentrations

**Fig. 8:**
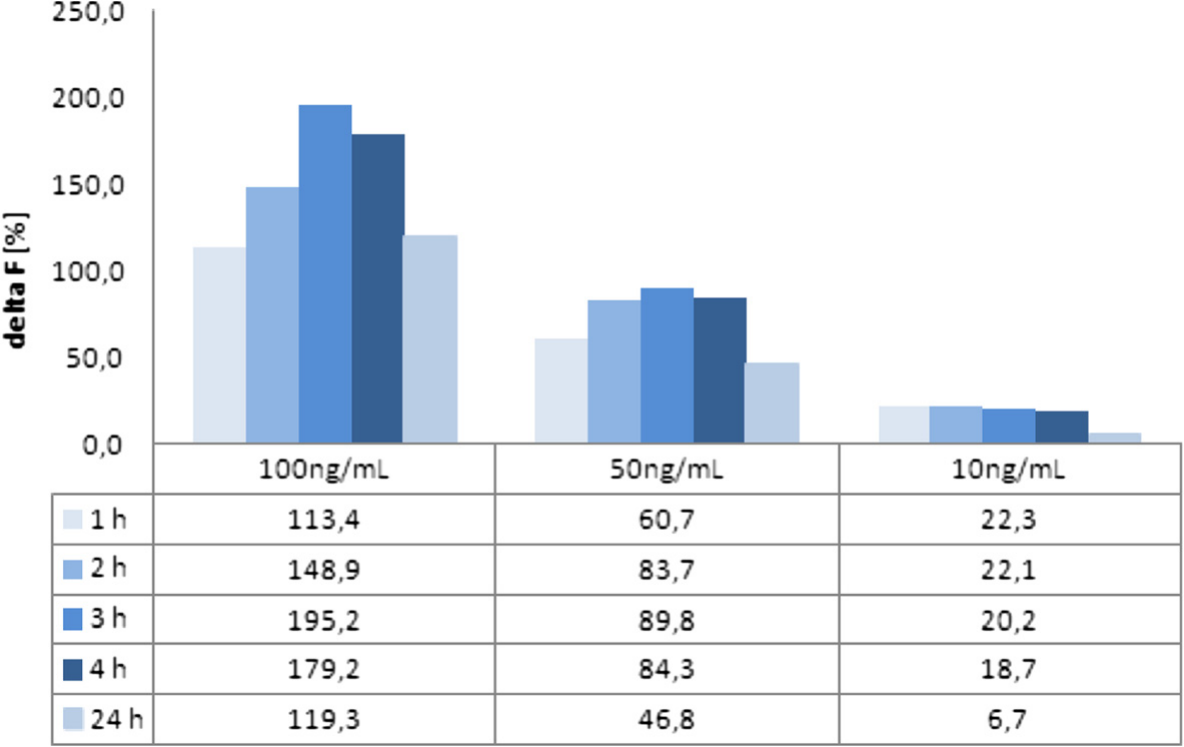
Different incubation times for proteinX using three different concentrations

Figures 6 and 7 show the results of different proteinY concentrations at different times. So, it is possible to define the necessary incubation time that allows stable results over a long period. For proteinY, an incubation time of 3 or 4 h seems to be sufficient, and for proteinX, a maximum value is reached after 3 h of incubation. These results show significant shorter incubation time compared to the ELISA incubation times of 13 to 15 h (Fig. 8).

Furthermore, Figs. 7 and 8 compare results of HTRF measured from 1 and 4 h and one measurement after 24 h which presents just little declines after 24 h. So, the HTRF assay is stable, analyzable, and evaluable over a long period of time without any bleaching effects.

### Comparison between ELISA and HTRF

Table 8 shows an overview about some summarized example measurements to show the final trends of both assays. Noticeable is the high difference between the linear ranges of the assays. ELISA has mostly a stable linearity between 14–1 ng/mL for proteinX and 10–1 ng/mL for proteinY. This range is approved by experiments to determine the accuracy and the precision. Inter-assay experiments are comparisons between the same sample and analyte, done on different microtiter plates, whereas intra-assays are between the same samples with different dilutions done on the same plate. The inter precision for HTRF assay seems to be higher. It can be influenced by the higher concentration range where the analyses are performed, but apart from this for both assays, it is below the limit of 20 %. For the accuracy, both assays are performed in the acceptable range of 100 ± 20 %, but the HTRF assay is just a little closer to 100 %. The intra-assay results of one example analyte produce for both assays nearly the same percentages of accuracy and precision.

**Table 9:** Comparison of ELISA and HTRF pros and cons

	ELISA	HTRF
Specificity	High	High
Universal	Yes	Yes
Homogenous	No	Yes
Time-resolved	No	Yes
Bleaching effects	Yes (30 min stable)	No
Hook effects	Not shown	Yes
Process time [h]	15–16 h	4–5 h
Material costs per well [€]	5–7 €	0.5–0.7 €
Automatization	No	Yes
Miniaturization	No	Yes
Linear range	36–1 ng/mL	100–2.5 ng/mL

Further possible positive/negative points are listed at Table 9 There, it is obvious that HTRF assay has some further advantages compared with ELISA. It is homogenous, so no washing steps are necessary that shortens the working and process time. The time-resolved measurement eliminates possible fluorescence background caused by other proteins, the analyte, or the donor antibody. Furthermore, ELISA reactions caused by consumed substrate are just stable for 30 min, whereas the HTRF fluorescence is constant for many hours and loses intensity just after 24 h, so any bleaching effects can be denied. Another positive point of HTRF is the possibility to use the assay with 384 well plates and small volumes of just 10 to 2.5 μL per analyte/antibody mix compared to 100 μL volume per analyte/antibody needed for ELISA 96-well plates. One possible disadvantage of HTRF is the limitation caused by the *Hook* effect that can negatively influence results of higher concentrated samples. With the limitation of the volume of analytes, this problem can be solved. All in all, these advantages are the reason why HTRF just needs 4–5h of process time (1 h for preparing samples/standard, 3–4 h for incubation), and ELISA needs much longer 15–16 h (12 h first antibody incubation, 1 h preparing samples/standard, 1 h blocking, 1 h second antibody incubation, washing, and developing). So, the analyzed sample per well costs, that are very interesting for the industry, can be reduced from 5–7 € for ELISA to 0.5–0.7 € for HTRF (calculated costs just contain material costs).

## Results

A constant increasing development of fragmented therapeutic monoclonal antibodies in the biomarker research and personalized medicine requires careful detection and quantification methods of antibody drug candidates. For many personalized therapeutic antibodies, no methods are available because of a lack of specific assay reagents. In this work, the development of a novel and very specific immuno assay for quantification of correct refolded Fab proteins by implementation of homogenous time-resolved fluorescence (HTRF) technology is done and compared with enzyme-linked immunosorbent assay (ELISA).

During this work, many results are collected to compare different immune assays and evaluate the benefits and disadvantages of all of them.

At first, Western blot is used to test different antibodies to find the specific one that can be used in further immune assays as a specific and universal detection antibody. The unlabeled monoclonal antibody of the company *Abcam* that is specific to the Hinge region of the Fab proteins combines high specificity and a generic applicability.

After a specific antibody is found, another immune assay is tested. ELISA has many possibilities of antibody-antigen combinations. Since *Abcam* is not labeled, a sandwich ELISA is done. By analyzing the linearity of the ELISA with two proteins, ranges with high regression coefficients (of about 0.99) are obtained. For proteinX, the range is between 36 and 1 ng/mL, and for proteinY, between 10 and 1 ng/mL. With these results, it can be seen that the linearity of the ELISA is restricted to a small range with low concentrations. The accuracy tested for this range is 88–99.4 % for proteinX and 80.9–95.9 % for proteinY in most cases. Furthermore, the precision of both proteins is tested in any kind of analysis by calculating the coefficient of variation (CV). For proteinX, the precision range is about 5.7–9.9 %, and for proteinY, 5.9–11.4 %. Accuracy and precision are for both proteins in the acceptable range defined by the FDA. Experiments, done with fermentation samples and different dilutions, show that the concentrations are comparable with little variations and coefficients of variation less than 15 %. Spike-in experiments to determine a range without matrix effects and to define stability are further tested parameters. ProteinX has acceptable recovery results between 40 and 1 ng/mL and coefficients of variation less than 20 % that means a high stability in this range which is just a little divergent to the linear range. The range of proteinY is 10 and 1 ng/mL and so exactly comparable with the linear range and also with low coefficients of variation about 15 %.

HTRF assay combines a universal homogenous immune assay with the properties of the FRET-effect and a time-resolved measurement. HTRF is used to evaluate the results of ELISA assay and to make an enhanced immune assay available. First, the linear range is determined; for the upper limit of proteinX, more than 100 ng/mL are useable and the linearity is stable until about 3.6–2.5 ng/mL. For proteinY, the range of 50–3.6 ng/mL is linear. So, with HTRF higher linear ranges are available, and also, the accuracy for proteinX of about 90–93 % and for proteinY of about 87.5–98.1 % lies in the favored guideline of 100 ± 15 %. The precision is just a little bit higher as for ELISA and lies between 5.7 and 16 % for proteinX and between 4.3 and 13.4 % for proteinY. By measuring samples with high concentrations (about 1 mg/mL), high variations between HTRF and ELISA assay can be observed, that can be caused by a great influence of high dilution factors and consequently dilution errors. With lower concentrations, a high reproducibility is possible (for example a sample about 100 ng/mL). Spike-in experiments show for proteinX acceptable recovery values of less than ±15 % of 100 %, even with concentrations of about 400 ng/mL, and for low concentrations of about 3–2 ng/mL, just little variations out of the favored guideline of ±20 %. The recovery of proteinY shows results in an acceptable range from 50 to 1 ng/mL with less than 20 % variation.

Finally, some problems like the *Hook* effect are tried to be solved by a combination of different volumes of analyte and antibodies to minimize the influence of *Hook* effect and to extend the available linear range of HTRF. While for proteinX no noticeable changes are observed, the useable linear concentration range of proteinY doubles.

Furthermore, a limitation of the used volumes until 2.5 μL of antibody mix and 2.5 μL of analyte shows little differences of the slope compared with the normal created curve (10 μL antibody mix + 10 μL protein), but also linear curves with acceptable *R*^2^ of 0.99. That initializes the facility to miniaturize the needed assay volumes.

The conclusion of the comparison of ELISA and HTRF assay is that both have some useable and irreplaceable abilities. For lower concentrated samples where high precision and accuracy are assumed and process times with volumes of about 100 μL per well are available, ELISA is suitable to bring a representative result. If an immune assay with minimal sample volume, the ability to measure 384 samples simultaneously, and less process time of about 4 h is preferred that shows good precision, linearity, and accuracy as well in high and low concentrations, the generic and homogenous HTRF assay should be the method of first choice.

Further, necessary steps would be to try to stabilize both methods even more to eliminate all possible influences like buffer effects or wrong antibody dilutions to get results without any kind of aberrations and errors. Therefore, many little facts like any kind of buffer or different dilutions of the antibody and also different plate types have to be tested if they influence the measurement. Automatization of both assays (for ELISA just the sample/standard dilution step could be automated) with a robot could be an advantage to avoid handling/pipette errors, to decrease working time, and to improve miniaturization. The current technology and paper conform with the recommendation of the “EPMA White Paper” [19].
